# Towards sustainable futures: development and validation of a Food Education Scale (FES) for upper elementary students in Taiwan

**DOI:** 10.1186/s12966-026-01925-w

**Published:** 2026-06-13

**Authors:** Li-Jiuan Lai, Tzuhui Angie Tseng

**Affiliations:** Department of Environmental and Cultural Resources, National Tsing Hua University, No.101, Section 2, Kuang-Fu Road, Hsinchu, 30013 Taiwan, R. O. C.

**Keywords:** Food literacy, Food education, Scale development, Elementary school students, Sustainability education

## Abstract

**Background:**

Food education has become an important policy priority in Taiwan following the enactment of the Food and Agriculture Education Act. However, empirically validated instruments for assessing food literacy among elementary school students remain limited. This study aimed to develop and validate a Food Education Scale (FES) for measuring food literacy among upper elementary school students in Taiwan.

**Methods:**

The scale was developed based on the six principles of Taiwan’s Food and Agriculture Education Act and Nutbeam’s (2000) framework of functional, interactive, and critical literacy. A two-dimensional framework integrating food education content and literacy competencies was constructed. Through expert review, pilot testing, and large-scale survey administration, a 36-item questionnaire was finalized. The formal survey included 400 upper elementary students from 12 schools across northern, central, southern, and eastern Taiwan. Reliability and construct validity were assessed using Cronbach’s α, exploratory factor analysis (EFA), and confirmatory factor analysis (CFA) to demonstrate satisfactory construct, convergent, and discriminant validity.

**Results:**

The results indicated that the FES demonstrated satisfactory psychometric properties. Internal consistency for all dimensions exceeded the recommended threshold (Cronbach’s α > 0.70), and both EFA and CFA supported the scale’s overall construct validity. In terms of performance patterns, students scored relatively higher in the dimensions related to cherishing food and reducing waste as well as food culture inheritance, while comparatively lower scores were observed in balanced dietary concepts and local production–local consumption. Differences in food literacy were also associated with school types, regional contexts, and family food practices.

**Conclusions:**

The Food Education Scale provides a validated instrument for assessing food literacy among upper elementary school students in Taiwan. The findings provide a validated, policy-aligned instrument for assessing children’s food literacy and offer practical guidance for curriculum design and sustainability-oriented food education policies.

## Background

The transformation of global food systems has become an urgent international issue. The United Nations’ *State of Food Security and Nutrition in the World* identifies several pathways for addressing food system challenges, including improving climate resilience, strengthening vulnerable populations, reducing the cost of nutritious foods, and reshaping food environments to support healthier diets. From a sustainability perspective, food waste further intensifies carbon emissions and climate risks. Consequently, the Sustainable Development Goals (SDGs), particularly Goals 1 and 2, emphasize eliminating poverty, ensuring food security, improving nutrition, and promoting sustainable agriculture. Within this global context, food literacy has gained increasing attention as a key competence that connects food systems, environmental sustainability, and public health. Food education policies have also evolved globally. The concept of Shokuiku (food education) originated in Japan and has become an influential framework for promoting healthy and sustainable dietary practices. The idea is commonly traced to the work of Japanese physician Sagen Ishizuka in the late nineteenth century, who emphasized that physical health, intellectual development, and moral cultivation are fundamentally connected to everyday dietary practices [1, 2]. His writings highlighted the importance of balanced diets, seasonal foods, and locally produced ingredients, laying the intellectual foundation for later food education movements.

In response to growing concerns about dietary transitions, declining food self-sufficiency, and increasing health problems, Japan institutionalized these ideas through the *Basic Act on Shokuiku*, enacted in 2005. This legislation aimed to strengthen public awareness of food systems, nutrition, food culture, and environmentally responsible consumption behaviors [2]. Since then, the concept of food education has expanded internationally and has been integrated into various educational and policy initiatives. Examples include farm-to-school programs in the United States, community-supported agriculture and the Slow Food movement in Europe, school-based food education initiatives in the United Kingdom, and sustainability-oriented food education programs in Nordic countries [2, 3]. These initiatives reflect a broader global trend that recognizes food education as an important pathway for linking dietary behaviors, public health, environmental sustainability, and cultural awareness. As a result, food education has increasingly been viewed as a key component of sustainable development and food system transformation.

In Taiwan, food education has gradually developed within both cultural and policy contexts. Traditional Chinese philosophy has long emphasized the relationship between agriculture, food, and social well-being. For example, Mencius argued that respecting agricultural seasons ensures food security and ecological balance [2]. Historical texts such as *The Book of Songs* and *The Rites of Zhou* also reflect agrarian traditions centered on food and agricultural practices. Contemporary food education in Taiwan integrates agricultural literacy, experiential learning, nutrition education, and sustainable lifestyles [3, 4]. The development of food education in Taiwan has progressed through four major phases—initiation (the 4-H movement in the 1950s), exploration, foundation building, and policy development—ultimately leading to the enactment of the Food and Agriculture Education Act in 2022 [5]. This legislation established a comprehensive framework for promoting food education nationwide. Current practices include government-led programs supporting local agricultural production and nutrition education, school-based initiatives integrating garden learning and local food curricula, and community-based activities organized by civil society organizations such as the Homemakers United Foundation and local farmers’ associations [6].

Food education is closely linked to sustainable development, which emphasizes meeting present needs without compromising the ability of future generations to meet their own needs [7]. In this context, food systems have increasingly been recognized as a key domain for sustainability education, connecting dietary practices, environmental impacts, and responsible consumption behaviors [8–10]. In Taiwan, multiple frameworks have been developed to conceptualize food education within sustainability and cultural contexts. These include models incorporating sustainability awareness, multicultural understanding, and low-carbon lifestyles [3], as well as expanded curricular approaches addressing environmental adaptation and sustainable diets [11]. Additional frameworks have identified thematic domains such as health, food safety, and agricultural sustainability [12], while more recent competency-based models emphasize autonomy, interaction, and mutual benefit across agricultural production, dietary health, and food culture [13].

Food literacy extends beyond traditional nutrition education or cooking instruction, emphasizing the interconnected relationships among food, health, society, and the environment [14, 15]. From a sustainability perspective, it integrates dietary knowledge, agricultural awareness, and environmental education through experiential and practice-oriented learning [16]. Accordingly, existing research conceptualizes food literacy as a multidimensional construct encompassing knowledge of personal health, food systems, and broader social, cultural, economic, environmental, and political contexts [9], with local studies in Taiwan further incorporating cultural awareness, behavioral practices, social responsibility, and sustainability concerns [3, 13, 17]. Theoretically, food literacy is commonly grounded in functional, interactive, and critical literacy models, as well as food-related practices such as planning, selecting, preparing, and consuming food [8, 18]. In addition, broader perspectives have expanded the concept to include ecological approaches, self-efficacy, community food security, and epistemological frameworks [19, 20]. Empirical studies have further identified key domains, attributes, and competencies, such as evaluating nutrition information and understanding long-term health implications [19, 21]. To operationalize these conceptualizations, various measurement instruments have been developed, including the Short Food Literacy Questionnaire (SFLQ) and the Self-Perceived Food Literacy Scale (SPFL), as well as frameworks integrating food system knowledge and sustainability-oriented competencies [21–24]. However, existing instruments often focus on specific competencies, such as nutrition knowledge or cooking skills, and remain limited in addressing children’s food literacy within integrated policy and sustainability contexts [21, 22]. Despite these advances, several key limitations remain. First, existing instruments tend to focus on specific competencies rather than providing an integrated framework linking food education, sustainability, and policy contexts. Second, validated tools specifically designed for children are still limited. Third, few studies have incorporated both educational policy frameworks and theoretical models into scale development. Accordingly, this study proposes a novel two-dimensional framework that integrates food education content with literacy competencies, thereby addressing these gaps.

To address this gap, this study develops and validates a multidimensional Food Education Scale (FES) for assessing food literacy among upper elementary school students in Taiwan. The scale is grounded in Nutbeam’s health literacy framework [25], the principles of Taiwan’s Food and Agriculture Education Act [26], and the United Nations Sustainable Development Goals (SDGs), thereby integrating theoretical and policy perspectives. Specifically, this study aims to (1) develop the FES, (2) examine its reliability as well as construct, convergent, and discriminant validity, and (3) explore patterns of food literacy among students.

## Methods

### Scale development procedure

The development of the scale in this study involved the following steps: (1) conducting a literature review; (2) inviting six experts from the fields of education, early childhood education, agriculture, and environmental studies to develop an appropriate pool of questionnaire items; (3) administering a pilot test with 30 children; (4) conducting the formal survey; and (5) examining the reliability and validity of the scale. In addition, during the formal survey, this study examined whether students’ background variables—such as gender, school region and type, family socioeconomic status (calculated based on parents’ education levels and occupational indices), participation in food and agricultural education courses or related activities, and daily life experiences—had significant effects on their food literacy scores.

### Research participants

Taiwan was divided into four regions—northern, central, southern, and eastern—and three types of schools (general, non-urban/non-mountainous, and remote) were selected from each region, resulting in a total of 12 schools across different counties and cities. Approximately 30–35 upper-grade elementary students were sampled from each school (a greater number of remote schools were included due to smaller student populations), yielding a total sample of 400 students who completed the questionnaire. Upper-grade elementary students (approximately 10–12 years old) were selected because previous studies suggest that children at this developmental stage generally possess the cognitive and reading abilities necessary to independently comprehend and complete structured questionnaires (Tseng et al., 2023) [27].

### Research instrument

The Children’s Food Literacy Scale was developed based on the six dimensions of Taiwan’s Food and Agricultural Education Act: (1) supporting and recognizing local agriculture, (2) cultivating balanced dietary concepts, (3) cherishing food and reducing waste, (4) inheriting and innovating food culture, (5) strengthening the connection between diet and agriculture, and (6) promoting sustainable local production and consumption. The items were designed based on the three categoroes of health literacy—functional, interactive, and critical literacy—thereby forming a two-dimensional conceptual framework that integrates food education content with literacy competencies [25] (Table 1). The scale aims to assess students’ levels of food literacy across these dimensions and to serve as a reference for improving educational programs and curriculum design. A five-point Likert scale was used, with responses ranging from 1 (“strongly disagree”) to 5 (“strongly agree”); higher scores indicate higher levels of food literacy.

**Table 1:** Two-dimensional framework of the Food Education Scale

Three categories Six dimensions	FC	IC	CC
SIA	1,2	3,4	5, 6
DBD	8,10	9	7, 11, 12
CRW	13,15, 18	16, 17	14
IFC	19, 21	20, 22, 24	23
DDA	26	--	25, 27, 28, 29, 30
PCA	31, 35	32, 34, 36	33

The scale items are listed in Tables 8 and 9. The three categories should be read vertically, while the six dimensions should be read horizontally. Six dimensions of Taiwan’s Food and Agricultural Education Act: Support and Identification with Local Agriculture(SIA); Developing a Balanced Dietary Concept (DBD); Cherishing Food and Reducing Waste (CRW); Inheriting and Innovating Food Culture(IFC); Deepening the Connection Between Diet and Agriculture(DDA); Local Production and Local Consumption for Sustainable Agriculture(PCS). Three dimensions of health literacy: Functional Category (FC); Interactive Category (IC); Critical Category (CC)

### Data analysis

Data were analyzed using SPSS 23.0 and AMOS 26.0 to examine the reliability and construct validity of the scale. Internal consistency was first assessed using Cronbach’s α. Exploratory factor analysis (EFA) was then conducted to identify the underlying factor structure, with items showing factor loadings below 0.5 removed to ensure clear construct representation [28]. Subsequently, confirmatory factor analysis (CFA) was performed to test the hypothesized measurement model derived from the six policy dimensions of Taiwan’s Food and Agriculture Education Act and Nutbeam’s (2000) health literacy framework. Model fit was evaluated using indices including the Root Mean Square Error of Approximation (RMSEA) and Comparative Fit Index (CFI). Due to sample size constraints, both EFA and CFA were conducted using the same dataset, a procedure commonly adopted in preliminary scale development studies. 

#### Expert validity

Content validity was assessed using the Content Validity Index (CVI) based on established procedures for evaluating expert agreement. Experts were asked to rate the importance of each item using a three-point scale (1 = not important, 2 = moderately important, 3 = important). The CVI for each item was calculated as the proportion of experts assigning a rating of 2 or 3, indicating that the item was considered relevant. When six to eight experts participate in the evaluation, a CVI value of 0.83 or higher is generally regarded as an acceptable threshold for content validity [29–32]. These criteria were applied to determine whether individual items should be retained, revised, or removed. In this study, the overall CVI was 0.93, indicating excellent expert validity. Items with variance greater than 0.30 were revised or deleted based on expert feedback. After refinement, the final version of the formal questionnaire consisted of 36 items, with six items for each of the six dimensions, for a total score of 180.

#### Pilot test statistical analysis

Before the formal survey, a pilot test was conducted with 30 upper-grade elementary students to evaluate the scale’s performance. The collected data were analyzed using factor analysis, reliability analysis, and item–total correlation analysis to revise, delete, or add items where necessary. The cumulative explained variance reached 75.7%, exceeding the commonly recommended threshold of 60% for exploratory factor analysis [28], indicating that the extracted factors adequately represented the underlying constructs. Most items showed factor loadings above 0.50, suggesting acceptable item–factor relationships; therefore, all items were retained for further analysis.

After defining high- and low-score groups, an independent samples t-test was conducted to determine whether significant differences existed in total scores. A significant t-value greater than 3 indicated good discriminative power of the scale. The results showed significant differences between groups, confirming strong discrimination. Correlation analysis further revealed that most item–total correlation coefficients exceeded 0.3, indicating good internal consistency. The reliability analysis using Cronbach’s α showed that all six dimensions had α values above 0.7, suggesting acceptable reliability.

## Results

### Demographic characteristics of the sample

Following ethical approval, 400 valid responses were collected between May and June 2023. The sample showed a relatively balanced distribution in gender (53.8% boys, 46.2% girls), geographic regions, and school types, with most students coming from two-parent households (83%) and middle to upper-middle socioeconomic backgrounds. Participation in food and agricultural education varied notably, with 44.2% of students reporting prior participation. Higher participation rates were observed in southern and eastern regions and in non-urban and remote schools. In contrast, direct exposure to agricultural environments was limited, with only 6.7% of students reporting frequent visits to farms. Regarding everyday food-related experiences, moderate engagement was observed, with 28% of students frequently accompanying family members to markets and 21.2% regularly participating in cooking activities. Overall, while the sample demonstrated balanced demographic characteristics, disparities in experiential learning opportunities and family food practices were evident.

### Scale validation/CFA discussion

The internal consistency reliability of the Food Literacy Scale was examined using Cronbach’s α coefficient to assess the internal coherence among items within each dimension. The Cronbach’s α values for all six dimensions of the Food and Agricultural Education Act and the three categories of food literacy exceeded 0.7, indicating acceptable reliability.

**Fig. 1:**
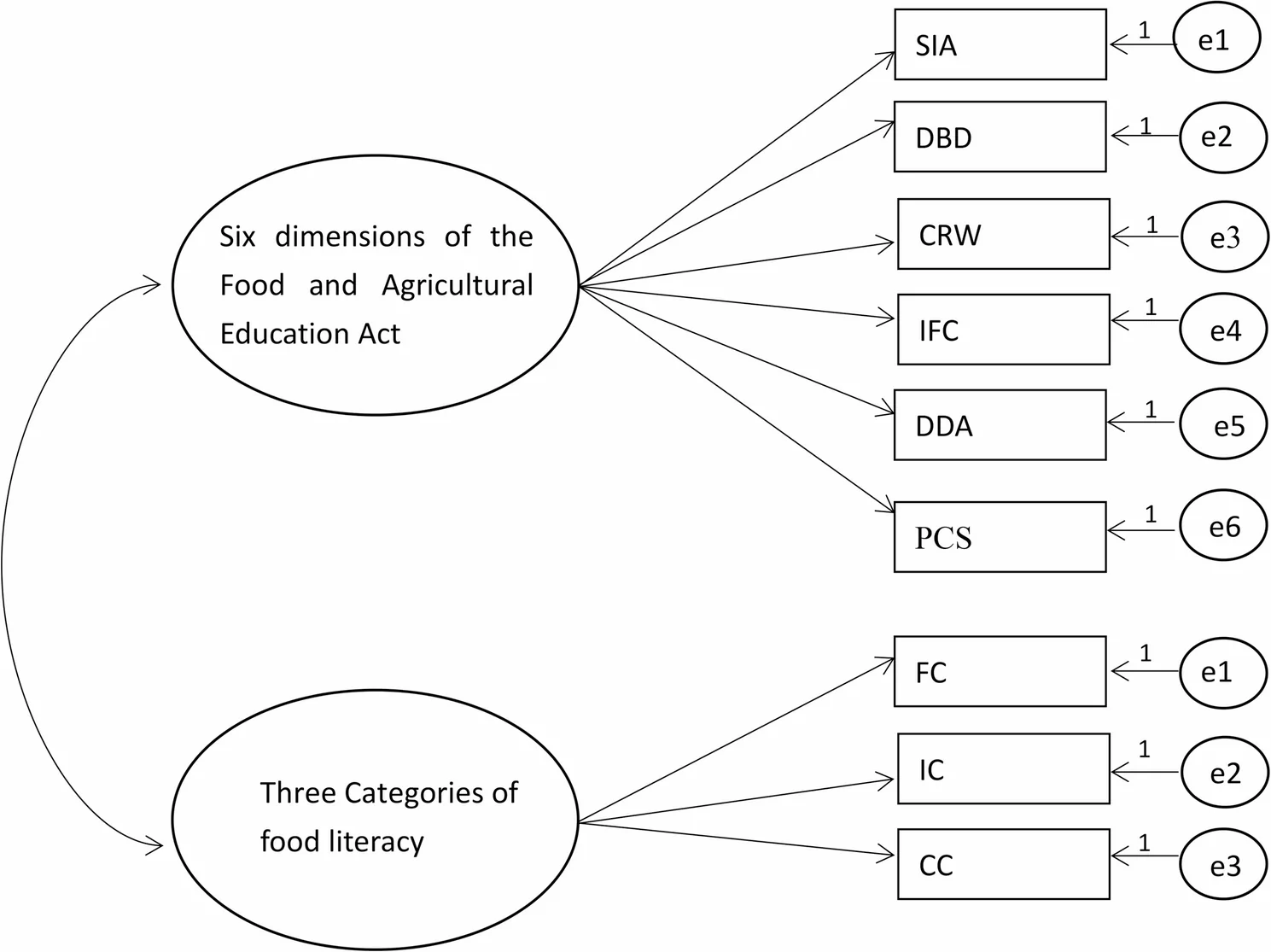
Confirmatory factor analysis model of the food literacy scale. Support and Identification with Local Agriculture(SIA); Developing a Balanced Dietary Concept (DBD); Cherishing Food and Reducing Waste (CRW); Inheriting and Innovating Food Culture(IFC); Deepening the Connection Between Diet and Agriculture(DDA); Local Production and Local Consumption for Sustainable Agriculture(PCS). Functional Category (FC); Interactive Category (IC); Critical Category (CC)

Confirmatory Factor Analysis (CFA) was employed to test the construct validity of the scale and to assess how well the theoretical model fit the observed data. Figure 1 presents the CFA model of the Food Literacy Scale, developed using AMOS 26.0 structural equation modeling software, illustrating the relationships between the observed indicators and latent variables. Although the RMSEA values appear relatively high, previous methodological studies have indicated that RMSEA may overestimate model misfit in models with small degrees of freedom [33]. Therefore, RMSEA should be interpreted together with other fit indices such as CFI, TLI, and standardized factor loadings.

#### Test of normality

To examine the normality of the data, this study first inspected the skewness and kurtosis values (Table 2). The results showed that, for the six dimensions of the Food and Agricultural Education Act, the absolute values of skewness ranged from 0.25 to 1.36 (< 2), and the absolute values of kurtosis ranged from 0.14 to 2.57 (< 7), meeting the criteria for univariate normality. Further examination of multivariate normality indicated that the Mardia’s multivariate kurtosis coefficient was 23.94, which was below the critical value of 48 [p × (*p* + 2), where p is the number of observed variables], demonstrating that the data satisfied the assumption of multivariate normality. Similarly, for the three categories of food literacy, the absolute values of skewness ranged from 0.36 to 0.68 (< 2), and the absolute values of kurtosis ranged from 0.26 to 0.66 (< 7), also meeting the criterion for univariate normality. The Mardia’s multivariate kurtosis coefficient was 2.53, which was lower than the critical value of 15 [p × (*p* + 2)], confirming that the data conformed to the assumption of multivariate normality.

**Table 2:** Normality tests for the food education literacy scale

	Variable	Min.	Max.	Skewness	Skewness *z*	Kurtosis	Kurtosis *z*
Six dimensions	**Support and Identification with Local Agriculture(SIA)**	1.00	5.00	− 0.72	-5.89	0.58	2.35
	**Developing a Balanced Dietary Concept (DBD)**	1.00	5.00	− 0.25	-2.04	− 0.35	-1.44
	**Cherishing Food and Reducing Waste (CRW)**	1.00	5.00	-1.36	-11.11	2.57	10.48
	**Inheriting and Innovating Food Culture(IFC)**	1.00	5.00	− 0.96	-7.80	1.03	4.18
	**Deepening the Connection Between Diet and Agriculture(DDA)**	1.00	5.00	− 0.35	-2.88	− 0.14	− 0.57
	**Local Production and Local Consumption for Sustainable Agriculture(PCS)**	1.00	5.00	− 0.06	− 0.52	− 0.49	-2.01
	**Multivariate (Coefficient)**					23.461	23.94
three categories	**Functional Category(FD)**	1.00	5.00	− 0.68	-5.56	0.66	2.67
	**Interactive Category (IG)**	1.00	5.00	− 0.42	-3.39	0.61	2.51
	**Critical Category (CG)**	1.00	5.00	− 0.36	-2.92	0.26	1.07
	**Multivariate (Coefficient)**					1.38	2.53

#### Examination of improper estimates

To ensure the rationality of the model estimation results, the error variances and standardized regression coefficients of each latent variable were examined. The results indicated that all error variances were positive and that the absolute values of all standardized estimates were below 0.95, suggesting that no improper estimates (Heywood cases) occurred in the model. The overall parameter estimation results of the model are presented in Table 3.

**Table 3:** Parameter estimates of the overall model

Parameter	Standardized Regression Weight	SE	t	EV	SMC
e1	0.79	.02	11.47	.26	.63
e2	0.72	.03	12.46	.40	.52
e3	0.76	.02	11.94	.24	.58
e4	0.74	.02	12.17	.25	.56
e5	0.83	.02	10.56	.21	.69
e6	0.78	.03	11.75	.30	.60
e7	0.90	.01	9.83	.10	.84
e8	0.93	.01	7.69	.06	.87
e9	0.92	.01	9.13	.08	.82

*SE* Standard Error, *EV* Error Variance, *SMC* Squared Multiple Correlation

#### Assessment of model fit

A higher level of model fit indicates greater usability and explanatory power of the model. According to structural equation modeling (SEM) conventions, goodness-of-fit indices can be classified into three categories: absolute fit indices, incremental fit indices, and parsimonious fit indices. In AMOS, a p-value greater than 0.05 from the chi-square (χ²) test is theoretically considered an indicator of good fit. However, because the chi-square statistic is highly sensitive to sample size, relying solely on this criterion is insufficient. Therefore, other fit indices should also be considered to provide a more comprehensive evaluation of model adequacy. If the overall model fit does not reach an acceptable level, researchers may consult the Modification Indices (M.I.) to guide model refinement. Generally, when the M.I. value exceeds 3.84 or 5, the parameter in question warrants further examination. Nevertheless, any decision to delete items or modify the model should be grounded in theoretical justification and the objectives of the study, rather than made purely on statistical grounds. A summary of the model fit indices for the Food Literacy Scale is presented in Tables 4 and 5. The RMSEA values exceeded commonly accepted thresholds, indicating limitations in model fit. Previous studies have suggested that RMSEA may overestimate model misfit in models with small degrees of freedom (Kenny et al., 2015) [33]. However, given the relatively high RMSEA values observed in this study, the model fit should be interpreted with caution. Other fit indices, including CFI, NFI, and standardized factor loadings, remained within acceptable ranges, providing partial support for the adequacy of the measurement model.

**Table 4:** Model fit indices for the six dimensions of the food and agriculture education act

Fit Index		Recommended Criterion	Result	Assessment
Absolute Fit Indices	χ^2^	Smaller is better	12.03(*p* = .001)	Meets criterion
	χ^2^/df	< 3	1.34	Meets criterion
	GFI	> .90	1.00	Meets criterion
	RMR	< .08	.00	Meets criterion
	RMSEA值	< .08	.19	Does not meet criterion
Incremental Fit Indices	AGFI	> .90	.72	Does not meet criterion
	NFI	> .90	1.00	Meets criterion
	CFI	> .90	1.00	Meets criterion
Parsimonious Fit Indices	PNFI	> .50	.54	Meets criterion
	PGFI	> .50	.54	Meets criterion

*GFI* Goodness-of-fit index, *RMR* Root mean square residual, *RMSEA* Root mean square error of approximation, *AGFI* Adjusted goodness-of-fit index, *NFI* Normed fit index, *CFI* Comparative fit index, *PNFI* Parsimonious normed fit index, *PGFI* Parsimonious goodness-of-fit index

**Table 5:** Model fit indices for the three categories of food literacy

Fit Index		Recommended Criterion |	Result	Assessment
Absolute Fit Indices	χ^2^	Smaller is better	31.54(0.001)	Does not meet criterion
	χ^2^/df	< 3	3.15	Does not meet criterion
	GFI	> .90	1.00	Meets criterion
	RMR	< .08	.00	Meets criterion
	RMSEA	< .08	.30	Does not meet criterion
Incremental Fit Indices	AGFI	> .90	1.00	Meets criterion
	NFI	> .90	1.00	Meets criterion
	CFI	> .90	1.00	Meets criterion
Parsimonious Fit Indices	PNFI	> .50	.69	Meets criterion
	PGFI	> .50	.51	Meets criterion

*GFI* Goodness-of-fit index, *RMR* Root mean square residual, *RMSEA* Root mean square error of approximation, *AGFI* Adjusted goodness-of-fit index, *NFI* Normed fit index, *CFI* Comparative fit index, *PNFI* Parsimonious normed fit index, *PGFI* Parsimonious goodness-of-fit index

#### Assessment of convergent validity

**Fig. 2:**
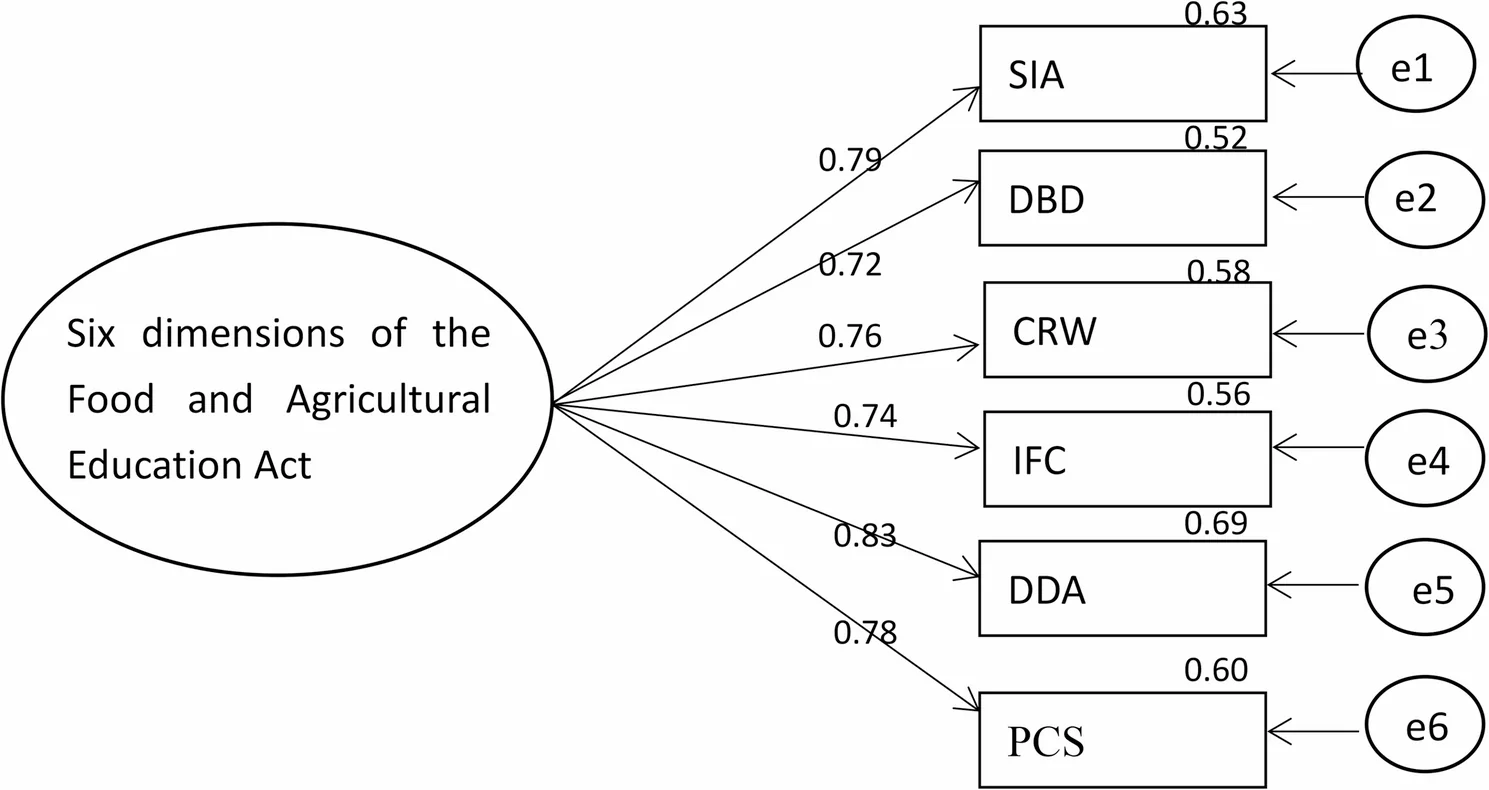
Convergent validity of the six dimensions of the food and agriculture education act. Support and Identification with Local Agriculture(SIA); Developing a Balanced Dietary Concept (DBD); Cherishing Food and Reducing Waste (CRW); Inheriting and Innovating Food Culture(IFC); Deepening the Connection Between Diet and Agriculture(DDA); Local Production and Local Consumption for Sustainable Agriculture(PCS)

**Fig. 3:**
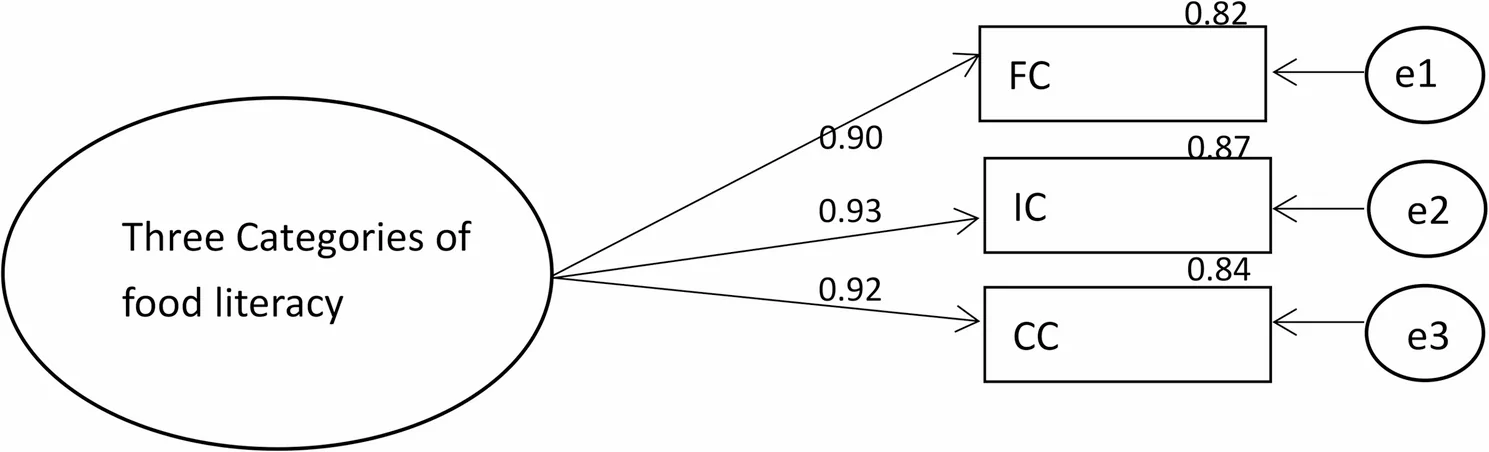
Convergent validity of the three categories of food literacy. Functional Category (FC); Interactive Category (IC); Critical Category (CC)

Convergent validity was evaluated using composite reliability (CR) and average variance extracted (AVE). Composite reliability reflects the internal consistency of a latent construct. Values of CR greater than 0.70 are generally considered acceptable for internal consistency, while AVE values above 0.50 indicate adequate convergent validity [28, 34]. The AVE measures the proportion of variance captured by a latent construct relative to the variance due to measurement error; a higher AVE indicates stronger convergent validity. Generally, an AVE greater than 0.50 supports adequate convergent validity. The results of this study indicated that the composite reliability for the six dimensions of the Food and Agricultural Education Act was 0.90 (> 0.60), and the average variance extracted was 0.60 (> 0.50), both meeting the accepted criteria for convergent validity (see Fig. 3). Similarly, for the three categories of food literacy, the composite reliability was 0.94 (> 0.60), and the average variance extracted was 0.84 (> 0.50), demonstrating good convergent validity (see Figs. 2 and 3). The relatively high AVE values indicate that the latent constructs account for a substantial proportion of variance in their observed indicators. This suggests that the scale items effectively represent the intended educational constructs, providing further support for the measurement quality of the Food Education Scale.

#### Assessment of discriminant validity

Discriminant validity was examined to determine whether the latent constructs were empirically distinct. Discriminant validity is supported when the square root of the average variance extracted (AVE) for each construct is greater than the correlations between that construct and other constructs, indicating that the construct shares more variance with its indicators than with other latent variables [34]. Results indicated that, for all six dimensions of the Food and Agriculture Education Act, the square roots of the AVEs were consistently greater than the corresponding inter-construct correlations, providing evidence of satisfactory discriminant validity (Table 6). Similarly, for the three categories of food literacy, the square roots of the AVEs surpassed the inter-factor correlations, further confirming adequate discriminant validity among the constructs (Table 7).

**Table 6:** Summary of discriminant validity for the six dimensions of the food and agriculture education act

Dimension	Number of Items	Correlation Coefficients					
		SIA	DBD	CRW	IFC	DDA	PCS
SIA	6	**(0.98)**					
DBD	6	0.76***	**(0.96)**				
CRW	6	0.85***	0.66***	**(0.99)**			
IFC	6	0.68***	0.56***	0.73***	**(1.17)**		
DDA	6	0.75***	0.68***	0.73***	0.86***	**(1.14)**	
PCS	6	0.70***	0.68***	0.61***	0.73***	0.95***	**(0.90)**

Off-diagonal values represent the correlations among latent variables; diagonal values (in bold) represent the square root of AVE

*SIA* Support and Identification with Local Agriculture, *DBD* Developing a Balanced Dietary Concept, *CRW* Cherishing Food and Reducing Waste, *IFC* Inheriting and Innovating Food Culture, *DDA* Deepening the Connection Between Diet and Agriculture, *PCS* Local Production and Local Consumption for Sustainable Agriculture

***p < .001

**Table 7:** Summary of discriminant validity for the three categories of food literacy

Categories	Number of Items	Correlation Coefficients		
		FC	IC	CC
FC	6	**(0.90)**		
IC	6	0.56***	**(0.85)**	
CC	6	0.52***	0.52***	**(0.95)**

Off-diagonal values represent the correlations among latent variables; diagonal values (in bold) represent the square root of AVE

*FC* Functional Category, *IC* Interactive Category, *CC* Critical Category

***p < .001

### Performance of students’ food literacy

#### Performance across the six dimensions of food education

Overall, students demonstrated moderate to high levels of food literacy across the six dimensions (Table 8), with an overall standardized score of 76.7 (138 out of a total possible score of 180). Higher performance was observed in “Cherishing Food and Reducing Waste” and “Inheriting and Innovating Food Culture,” whereas comparatively lower scores were found in “Developing a Balanced Dietary Concept” and “Local Production and Local Consumption for Sustainable Agriculture.” At the item level, students showed strong awareness of environmentally friendly agricultural practices and food-related cultural knowledge, while lower scores were associated with behavioral items such as reducing sugary beverage consumption and understanding local food systems. These findings suggest that while students possess foundational knowledge and positive attitudes toward food and sustainability, gaps remain in behavior-oriented and sustainability-related competencies, particularly in translating knowledge into everyday food-related practices.

**Table 8:** Scores of each item across the six dimensions of the food and agriculture education act

Dimension	Item	M	SD	Dimension Total
Support and Identification with Local Agriculture(SIA)	1. I pay attention to whether the food I eat is produced locally or imported.	3.65	1.18	23.45
	2. I know what agricultural products are produced in my local area.	3.60	1.21	
	3. I understand how foods such as eggs or milk travel from farms to our dining tables.	3.87	1.20	
	4. I support the use of local ingredients in school lunch programs.	3.98	1.13	
	5. I know what “food miles” are, and I agree with choosing locally produced foods, seasonal ingredients, and environmentally friendly products with shorter transportation distances.	3.89	1.11	
	6. I support managing farmland and cultivating crops using eco-friendly and non-toxic practices	4.46	0.91	
Developing a Balanced Dietary Concept (DBD);	7. I refuse sugary processed beverages when offered by classmates or friends” had the lowest score	3.02	1.31	21.37
	8. I eat a balanced variety of foods, including grains, fish, meat, vegetables, fruits, milk, etc.	4.01	1.11	
	9. I pay attention to whether I consume too many processed foods each day.	3.47	1.26	
	10.I know what food additives are, such as emulsifiers, colorants, flavor enhancers, spices, preservatives, and softening agent	4.02	1.16	
	11. I prefer drinking water from my own bottle rather than store-bought beverages.	3.34	1.33	
	12. I think fast foods such as hamburgers, fries, and fried chicken are unhealthy.	3.51	1.28	
Cherishing Food and Reducing Waste (CRW)	13. I check the expiration date when purchasing food products	4.44	0.99	25.05
	14. I know the correct methods for keeping food fresh.	4.15	1.05	
	15. I pay attention to expiration dates of food stored in the refrigerator or pantry and try to consume them before they expire.	4.22	1.08	
	16. I often share food or snacks with my classmates.	3.66	1.26	
	17. I cherish food.	4.29	0.97	
	18. I know the correct recycling categories for food packaging (e.g., metal cans, plastics, paper).	4.29	1.02	
Inheriting and Innovating Food Culture(IFC)	19. I know about the food cultures from my parents’ or grandparents’ hometowns (e.g., Hakka, Minnan, Vietnamese, Indonesian).	4.14	1.13	24.67
	20. I feel that my daily diet is becoming increasingly internationalized (e.g., Western, Japanese, Korean, Southeast Asian foods	3.73	1.20	
	21. I know the traditional foods associated with different festivals, such as tangyuan for the Winter Solstice, mooncakes for the Mid-Autumn Festival, zongzi for the Dragon Boat Festival, and yuanxiao for the Lantern Festival	4.45	0.94	
	22. I am willing to try different local Taiwanese foods (e.g., Taichung meatballs, Hsinchu meatballs, Hakka rice foods).	4.31	0.99	
	23. I like trying foods from different countries (e.g., Vietnamese, Thai, Korean).	4.03	1.18	
	24. When eating dinner with my family, we often talk about our daily lives.	4.01	1.18	
Deepening the Connection Between Diet and Agriculture(DDA)	25. I know that climate change affects food production.	4.28	0.99	22.68
	26. I know which fruits and vegetables are in season.	3.80	1.17	
	27. I may choose to avoid or reduce eating certain fish species that are overharvested.	3.64	1.26	
	28. I encourage my family to buy humanely raised eggs even if they are more expensive.	3.50	1.25	
	29. I agree that preferring meat consumption contributes to global warming.	3.45	1.35	
	30. I know what genetically modified foods are.	4.01	1.16	
Local Production and Local Consumption for Sustainable Agriculture(PCS)	31. I take the initiative to learn whether the foods my family buys are locally produced or imported.	3.64	1.24	21.35
	32. Most of the foods I eat daily are produced in Taiwan	3.96	1.06	
	33. I often go to convenience stores with classmates to buy snacks	3.17	1.42	
	34. My family likes to buy local produce from farmers’ associations or local farmers’ markets.	3.43	1.27	
	35. I have heard about Taiwan’s “Three Labels and One QR Code” food certification system (CAS, TAP traceability, organic certification, and production traceability QR code).	3.46	1.37	
	36. I share the benefits of purchasing pesticide-free and chemical-free food products with my family.	3.69	1.28	

### Performance across the three categories of food literacy

Across the three categories (Table 9), “functional” literacy showed the highest performance, followed by “interactive” and “critical” literacy, with an overall standardized score of 77.2. Students demonstrated relatively strong knowledge of food-related concepts and cultural practices, whereas lower performance was observed in behavior-oriented and decision-making items, particularly those related to healthy dietary choices and sustainable consumption. These results indicate that although basic knowledge is well established, the development of higher-order competencies—such as critical evaluation and behavioral application—remains comparatively limited.

**Table 9:** Item scores for the three categories of food literacy

Dimension	Item	M	SD	Dimension Total	
Functional Category (FC)	1. I pay attention to whether the food I eat is produced locally or imported.	3.65	1.18	47.72	
	2. I know what agricultural products are produced in my local area.	3.60	1.21		
	8. I eat a balanced variety of foods, including grains, fish, meat, vegetables, fruits, milk, etc.	4.01	1.11		
	10.I know what food additives are, such as emulsifiers, colorants, flavor enhancers, spices, preservatives, and softening agent	4.02	1.16		
	13. I check the expiration date when purchasing food products	4.44	0.99		
	15. I pay attention to expiration dates of food stored in the refrigerator or pantry and try to consume them before they expire.	4.22	1.08		
	18. I know the correct recycling categories for food packaging (e.g., metal cans, plastics, paper)	4.29	1.02		
	19. I know about the food cultures from my parents’ or grandparents’ hometowns (e.g., Hakka, Minnan, Vietnamese, Indonesian).	4.14	1.13		
	21. I know the traditional foods associated with different festivals, such as tangyuan for the Winter Solstice, mooncakes for the Mid-Autumn Festival, zongzi for the Dragon Boat Festival, and yuanxiao for the Lantern Festival	4.45	0.94		
	26. I know which fruits and vegetables are in season.	3.80	1.17		
	31. I take the initiative to learn whether the foods my family buys are locally produced or imported.	3.64	1.24		
	35. I have heard about Taiwan’s “Three Labels and One QR Code” food certification system (CAS, TAP traceability, organic certification, and production traceability QR code).	3.46	1.37		
Interactive Category (IC)	3. I understand how foods such as eggs or milk travel from farms to our dining tables.	3.87	1.20		46.57
	4. I support the use of local ingredients in school lunch programs.	3.98	1.13		
	9. I pay attention to whether I consume too many processed foods each day.	3.47	1.26		
	16. I often share food or snacks with my classmates.	3.66	1.26		
	17. I cherish food.	4.29	0.97		
	20. I feel that my daily diet is becoming increasingly internationalized (e.g., Western, Japanese, Korean, Southeast Asian foods	3.73	1.20		
	2. I am willing to try different local Taiwanese foods (e.g., Taichung meatballs, Hsinchu meatballs, Hakka rice foods).	4.31	0.99		
	24. When eating dinner with my family, we often talk about our daily lives.	4.01	1.18		
	32. Most of the foods I eat daily are produced in Taiwan	3.96	1.06		
	33. I often go to convenience stores with classmates to buy snacks	3.17	1.42		
	34. My family likes to buy local produce from farmers’ associations or local farmers’ markets.	3.43	1.27		
	36. I share the benefits of purchasing pesticide-free and chemical-free food products with my family.	3.69	1.28		
Critical Category (CC)	5. I know what “food miles” are, and I agree with choosing locally produced foods, seasonal ingredients, and environmentally friendly products with shorter transportation distances.	3.89	1.11		45.28
	6. I support managing farmland and cultivating crops using eco-friendly and non-toxic practices	4.46	0.91		
	7. I refuse sugary processed beverages when offered by classmates or friends” had the lowest score	3.02	1.31		
	11. I prefer drinking water from my own bottle rather than store-bought beverages.	3.34	1.33		
	12. I think fast foods such as hamburgers, fries, and fried chicken are unhealthy.	3.51	1.28		
	14. I know the correct methods for keeping food fresh.	4.15	1.05		
	23. I like trying foods from different countries (e.g., Vietnamese, Thai, Korean).	4.03	1.18		
	25. I know that climate change affects food production.	4.28	0.99		
	27. I may choose to avoid or reduce eating certain fish species that are overharvested.	3.64	1.26		
	28. I encourage my family to buy humanely raised eggs even if they are more expensive.	3.50	1.25		
	29. I agree that preferring meat consumption contributes to global warming.	3.45	1.35		
	30. I know what genetically modified foods are.	4.01	1.16		

### Differences in food and agriculture education act and food literacy across participant characteristics

#### Examining differences by school type

A one-way ANOVA was conducted to examine differences among “general”, “non-mountain–non-city (sub-urban)”, and “remote” school types in both the six dimensions of the Food and Agriculture Education Act and the three categories of food literacy. Significant group differences were found across all dimensions and categories (*p* < .05). Post hoc Scheffé tests showed that students in non-mountain–non-city schools consistently outperformed those in general and remote schools in the dimensions of “supporting local agriculture”, “developing balanced dietary concepts”, and “reducing food waste”; they also scored higher than remote schools in inheriting and innovating food culture and in local production for sustainable agriculture. Remote schools scored lower than both comparison groups in deepening the connection between diet and agriculture. For the three categories of food literacy, “sub-urban schools” again demonstrated higher “functional”, “interactive”, and “critical” literacy than both general and remote schools, while general schools outperformed remote schools in “critical” literacy. “Remote schools” showed the lowest scores across all three categories, suggesting potential disparities related to limited instructional resources and support.

#### Differences by gender, family socioeconomic status, food–agriculture learning experiences, and daily food-related practices

Independent-samples *t-tests* were conducted to examine gender differences in food and agriculture education and food literacy. Results showed a significant difference only in the dimension of “Inheriting and Innovating Food Culture,” *t*(398) = − 2.61, *p* < .05, *d* = 0.26, with girls scoring higher (*M* = 4.21, *SD* = 0.65) than boys (*M* = 4.02, *SD* = 0.81). For the three categories of food literacy, gender differences emerged only in interactive literacy, *t*(398) = 0.86, *p* < .05, *d* = 0.09, with girls again outperforming boys (*M* = 3.83, *SD* = 0.63 vs. *M* = 3.77, *SD* = 0.72). These findings are largely consistent with previous research indicating no significant gender differences in general food literacy and demonstrating measurement invariance across gender, suggesting that sustainable food literacy levels are generally comparable between boys and girls [24, 35].

Family socioeconomic status (parents’ education and occupation) did not yield significant differences across the six dimensions of the Food and Agriculture Education Act or the three categories of food literacy. This result is consistent with prior studies indicating that education level is not significantly associated with food literacy [21, 24]. The present study further suggests that overall socioeconomic status also does not significantly influence food literacy.

Experience with food–agriculture curricula resulted in a significant difference only in the dimension of “*Deepening the Connection between Diet and Agriculture*,” *t* (398) = 2.06, *p* < .05, *d* = 0.21, with students who had taken such courses scoring higher. No significant differences were found for the three food literacy categories. In contrast, students who frequently visited farms or agricultural/animal husbandry sites showed significant differences across almost all dimensions (except “*Cherishing Food and Reducing Waste*”) and across all three literacy categories, with frequent visitors scoring higher. These results indicate that agricultural tourism may exert a stronger influence on children’s food literacy than formal food–agriculture coursework, supporting evidence from prior studies showing that farm experiences shape children’s dietary behaviors and foster responsible consumption [27, 36].

Finally, everyday family food practices showed meaningful effects. Frequency of accompanying family members to markets was significantly associated with all dimensions except “Developing a Balanced Dietary Concept,” and both shopping with family and cooking with family members were linked to higher scores across all dimensions and all three literacy categories. These findings echo prior research demonstrating that shopping enhances children’s understanding of food [37] and that shopping and cooking with family effectively build children’s health literacy [38].

## Discussion

A Food Education Scale (FES) for upper elementary school students in Taiwan was developed and validated based on the six principles of the Food and Agriculture Education Act and Nutbeam’s [25] framework of functional, interactive, and critical literacy. The results demonstrated satisfactory reliability and construct validity, indicating that the proposed scale provides a robust instrument for measuring children’s food literacy. These findings support previous research suggesting that food literacy should be understood as a multidimensional construct encompassing knowledge, attitudes, and behavioral competencies related to food systems and sustainability [8, 9].

The psychometric analyses showed strong internal consistency across all six dimensions, with Cronbach’s α values exceeding 0.70 [39]. Confirmatory factor analysis further supported the two-dimensional framework integrating food education content with literacy competencies. This structure is consistent with previous studies conceptualizing food literacy as including functional knowledge, interactive learning, and critical awareness of food systems [19, 40]. The findings also demonstrate that integrating national policy frameworks with international food literacy models can generate culturally relevant assessment tools.

Regarding student performance, higher scores were observed in “*Cherishing Food and Reducing Waste (CRW)*” and “*Inheriting and Innovating Food Culture (IFC)*.” These results may reflect Taiwan’s cultural traditions emphasizing respect for food and family dining practices, which have been shown to shape children’s food-related attitudes and behaviors [37]. In contrast, lower scores were found in “*Developing a Balanced Dietary Concept (DBD)*” and “*Local Production and Local Consumption for Sustainable Agriculture(PCS)”*, echoing international research indicating that children often have limited understanding of sustainable food systems and healthy dietary decision-making [40, 41].

The results also highlight the importance of experiential learning. Students who frequently visited farms or agricultural environments showed significantly higher food literacy across most dimensions. This finding aligns with previous studies indicating that farm-based experiences can enhance children’s understanding of food systems and promote responsible consumption behaviors [27, 36]. Compared with traditional classroom instruction, experiential learning appears to offer more meaningful opportunities for connecting food production, environmental sustainability, and personal dietary practices.

Family interactions were another influential factor. Activities such as shopping for food and cooking with family members were associated with higher food literacy scores. These findings are consistent with prior research showing that family food environments strongly influence children’s food knowledge and dietary behaviors [37, 38]. Such everyday experiences may reinforce food-related knowledge and foster more critical awareness of food choices.

Regional and school-level differences were also observed. Students from sub-urban schools demonstrated higher food literacy levels than those from general or remote schools. These disparities may reflect differences in educational resources and access to experiential learning opportunities. Similar patterns have been reported in previous studies examining the influence of socioeconomic and environmental factors on children’s food literacy and dietary behaviors [41, 42].

From an educational perspective, the findings highlight the need to strengthen curriculum design by integrating behavior-oriented and sustainability-focused components, particularly in areas such as healthy dietary decision-making and local food systems. From a policy perspective, the results support the implementation of experiential and context-based food education programs that connect students’ daily lives with broader food system and sustainability issues. Overall, the findings underscore the importance of integrating food education with sustainability-oriented learning approaches. Multidisciplinary and experiential learning strategies have been shown to enhance children’s understanding of sustainable food systems and responsible consumption practices [10, 43]. The scale developed in this study provides a validated instrument that can support the evaluation of food education programs and inform future curriculum development.

## Conclusion

This study developed and validated a Food Education Scale (FES) for assessing food literacy among upper elementary school students in Taiwan. Grounded in the six principles of Taiwan’s Food and Agriculture Education Act [8] and Nutbeam’s [25] health literacy framework, the scale integrates food education content with literacy competencies within a two-dimensional model. The results demonstrated strong internal consistency and satisfactory construct, convergent, and discriminant validity, confirming the reliability of the instrument.

The findings indicate that Taiwanese elementary students generally demonstrate moderate to high levels of food literacy, particularly in food conservation and cultural awareness. However, lower scores in balanced dietary concepts and sustainable food consumption highlight areas requiring further educational attention. These results suggest that food education programs should strengthen nutrition literacy and sustainability-oriented learning. The study also highlights the importance of experiential learning and family engagement. Activities such as visiting farms, participating in agricultural experiences, cooking with family members, and engaging in everyday food practices were associated with higher food literacy levels.

Although several group differences were statistically significant, the effect sizes were relatively small, suggesting that the practical differences in students’ food literacy may be modest. This indicates that while certain contextual factors may influence food literacy development, the overall competencies among students are relatively comparable across groups. Several limitations should be acknowledged. First, the data were collected using self-report questionnaires, which may be subject to response bias. Second, the cross-sectional design limits the ability to draw causal inferences regarding the development of students’ food literacy. Longitudinal studies may provide deeper insights into how food literacy evolves over time. Third, this study was conducted within the context of Taiwan’s Food and Agriculture Education Act; therefore, the findings may reflect specific cultural and policy contexts. Future research could examine the applicability of the scale in different educational and cultural settings. In addition, because students were sampled from multiple schools, future studies may consider applying multilevel modeling to further examine potential school-level influences.

Overall, the Food Education Scale developed in this study provides a practical tool for evaluating food education outcomes and supporting evidence-based curriculum design. The scale may also facilitate future comparative research on food education across different countries.

## Data Availability

The data supporting the findings of this study are available for review upon reasonable request but are not publicly shared due to participant confidentiality and restrictions stipulated by the Institutional Review Board approval (IRB No. 11204HT042). Researchers who wish to access the data for verification purposes may contact the corresponding author. Access will be granted under conditions that ensure the protection of participant privacy and compliance with ethical guidelines.

## Abbreviations

FES: Food Education Scale
SDGs: Sustainable Development Goals
SFLQ: Short Food Literacy Questionnaire
SPFL: Self-Perceived Food Literacy Scale
SES: Socioeconomic Status
SPSS: Statistical Package for the Social Sciences
AMOS: Analysis of Moment Structures
EFA: Exploratory Factor Analysis
CFA: Confirmatory Factor Analysis
SEM: Structural Equation Modeling
CVI: Content Validity Index
CR: Composite Reliability
AVE: Average Variance Extracted
RMSEA: Root Mean Square Error of Approximation
CFI: Comparative Fit Index
GFI: Goodness-of-Fit Index
AGFI: Adjusted Goodness-of-Fit Index
RMR: Root Mean Square Residual
NFI: Normed Fit Index
PNFI: Parsimonious Normed Fit Index
PGFI: Parsimonious Goodness-of-Fit Index
SE: Standard Error
EV: Error Variance
SMC: Squared Multiple Correlation
SIA: Support and Identification with Local Agriculture
DBD: Developing a Balanced Dietary Concept
CRW: Cherishing Food and Reducing Waste
IFC: Inheriting and Innovating Food Culture
DDA: Deepening the Connection Between Diet and Agriculture
PCS: Local Production and Local Consumption for Sustainable Agriculture
FC: Functional Category
IC: Interactive Category
CC: Critical Category
